# Complete plastome of an endemic fern species from China: *Neocheiropteris palmatopedata* (Polypodiaceae)

**DOI:** 10.1080/23802359.2018.1532840

**Published:** 2019-07-12

**Authors:** Xin-Yu Du, Jin-Mei Lu, Shu-Gang Lu, De-Zhu Li

**Affiliations:** a School of Life Sciences, Yunnan University, Kunming, China;; b Germplasm Bank of Wild Species, Kunming Institute of Botany, Chinese Academy of Sciences, Kunming, China;; c Kunming College of Life Science, University of Chinese Academy of Sciences, Kunming, China

**Keywords:** Plastome, fern, *Neocheiropteris palmatopedata*

## Abstract

*Neocheiropteris palmatopedata* (Baker) Christ is an endangered fern species endemic to southwest China. In this study, we sequenced the complete plastid genome of *N. palmatopedata*. The gene order and structure of the *N. palmatopedata* plastome are similar to those published plastomes in Polypodiales. The complete plastome is 153,344 bp in length, and the GC content is 42.1%. The plastome comprises 113 unique genes (83 protein-coding genes, 29 tRNA genes and four rRNA genes).

*Neocheiropteris palmatopedata* (Baker) Christ is an endangered fern species, and endemic to southwest China. This species only distributed in subtropical or tropical dry forests, and subtropical or tropical moist lowland forests in Yunnan, Guizhou and Sichuan provinces. *Neocheiropteris palmatopedata* is a beautiful and ornamental fern in gardening, and is also used as a folk medicine to treat astriction, chronic gastritis, laryngopharyngitis, ovarian dropsy, and rheumatism (Wu 1990). This species was listed as Endangered (A2c) according to the IUCN Red List Categories and Criteria (https://www.iucnredlist.org). The distribution area of *N. palmatopedata* is extremely fragmented and increasingly reduced because of human interference and habitat destruction, thereby calling for appropriate conservation strategies to save the genetic diversity of this species.

In this study, we reported the complete plastid genome of *N. palmatopedata*. Fresh leaf material was collected from Kunming Botanical Garden and the voucher specimen was deposited at Herbarium, Kunming Institute of Botany, CAS (KUN, collection number: FB663). An improved method for plastid DNA purification by high ionic strength in combination with low pH (3.6) buffer (Gong et al. 1994; Jansen et al. 2005) was used to extract DNA. Illumina Solexa platform (Illumina, San Diego, CA) was used to sequence paired-end reads of the 500 bp insert-size libraries, and the length of the reads is 100 bp. Library preparation and sequencing were conducted at BGI Genomics Co., Ltd. (Shenzhen, China). *De novo* assembly was constructed with SPAdes 3.9.1 (Bankevich et al. 2012), GetOrganelle (Jin et al. 2018) was also used to improve efficiency in *de novo* assembly. Reference guided connecting was subsequently conducted using Bandage 0.8.1 (Wick et al. 2015) and Geneious 9.1.4 (Biomatters Ltd., Auckland, New Zealand), *Lepisorus clathratus* (NC_035739) was used as reference for assembling and annotation. The plastome was annotated in Geneious, start and stop codons of protein-coding genes were adjusted manually if necessary. The plastome sequence was deposited to GenBank (accession number: MH707375).

The gene order and structure of the *N. palmatopedata* plastome are similar to those published plastomes in Polypodiales (e.g. Wolf et al. 2010; Grewe et al. 2013). The complete plastome is 153,344 bp in length and consists of a large single-copy (LSC) region of 80,709 bp and a small single-copy (SSC) region of 21,761 bp, which are separated by two inverted repeats (IR) of 24,937 bp. The plastome comprises 113 unique genes (83 protein-coding genes, 29 tRNA genes and four rRNA genes). The GC content of the plastome is 42.1%. The average reads coverage of the sequencing is 893×. To identify the phylogenetic positions of *N. palmatopedata*, thirteen plastomes with one of the IR regions was excluded were aligned using MAFFT (Katoh et al. 2005), and a maximum-likelihood phylogenetic tree was constructed by RAxML (Stamatakis 2014) with 1000 rapid bootstrap replicates and the GTRGAMMA substitution model (Figure 1). The sister relationship between *N. palmatopedata* and *Lepisorus clathratus* is supported by 100% bootstrap value (Figure 1). However, more complete plastome data from Polypodiaceae are needed to confirm its phylogenetic position.

**Figure 1.**
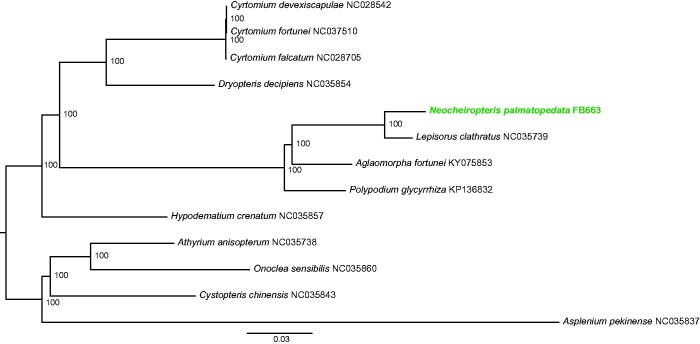
A maximum-likelihood tree based on 13 plastomes and constructed in RAxML. Bootstrap support values are shown on the nodes.
